# Two-third of inmates were depressed among HIV positive prisoners at central prison (Kaliti), Addis Ababa, Ethiopia

**DOI:** 10.1186/s13104-019-4216-1

**Published:** 2019-03-25

**Authors:** Meselu Getaneh, Mebratu Mitiku Reta, Dawit Assefa, Zegeye Yohannis, Demeke Demilew

**Affiliations:** 1 Department of Psychiatry, University of Gondar and Amanuel Specialized Mental Health, Addis Ababa, Ethiopia; 2 Department of Internal Medicine, School of Medicine, University of Gondar, Gondar, Ethiopia; 3 Amanuel Specialized Mental Health Hospital, Addis Ababa, Ethiopia; 4 Department of Psychiatry, School of Medicine, University of Gondar, Gondar, Ethiopia

**Keywords:** Depression, HIV/ADIS, Prison, Ethiopia

## Abstract

**Objective:**

Depression is the most frequently observed psychiatric disorder among HIV positives and it is becoming worse in prisoners. The aim this study was to assess prevalence and associated factors of depression among HIV positive prisoners at central prison (Kaliti) Addis Ababa.

**Result:**

The prevalence of depression was found to be 66.5% (95% CI 62, 71). Primary education [AOR = 4.17, 95% CI (1.648–10.483)], perceived stigma [AOR = 3.88, 95% CI (2.08–7.25)], history of chronic illness [AOR = 2.88, 95% CI (1.34–6.17)] and WHO clinical stage II [AOR = 2.47, 95% CI (1.19–5.12)] and length of stay in prison 4–6 years[AOR = 2.27, 95% CI (1.22–4.23)] and ≥ 10 years [AOR = 3.53, 95% CI (1.15–10.85)] were factors associated with depression. This study indicated that prevalence of depression among HIV positive prisoners in Addis Ababa prison was high. Educational status, perceived stigma, history of chronic illness, WHO clinical stage and length of stay in prison were factors associated with depression. Establishing psychiatry care to screen and manage mental health disorders at the prison is needed. Special attention has to be given for those with primary in education, advanced disease, perceived stigma, and history of chronic illness and stayed many years in prison.

## Introduction

According to WHO report, 28% of global burden of disease is due to neuropsychiatric disorder and one-third of it is caused by depression [1]. Depression is common in HIV infected patients and it affects the course of the disease [2, 3]. Depression and HIV/AIDS are projected to be the world`s two leading causes of disability by 2030 [4]. Diagnosis and treatment of depression in HIV/AIDS patients is complicated due to interactions between the disease conditions as well as drug–drug interaction between ART and anti depressants [5]. In 2017, the number of people living with HIV in the world was 36.9 million and 1·8 million were new infections and there were 940,000 HIV/AIDS related deaths in the same year [6]. According to 2016 Ethiopian DHS, the prevalence of HIV was estimated to be 1.1% [7]. However, International data indicates that HIV prevalence among incarcerated persons is 5 times greater than the general population [8]. Previous studies indicated that prevalence of depression among prisoner living with HIV/AIDS was higher than general population, 55.5% in USA, 31.2% in Taiwan, 55.4% In Durban South Africa) [9–11]. People living with HIV/AIDS are more stressed with long-term discomfort, physical deterioration and economic dependence [12]. These all my lead them to develop depression. In the context of HIV/AIDS, depression can affect the health seeking behavior and causes poor treatment adherence that leads to treatment failure and rapid progression to AIDS and death [13]. In addition, depression causes decreased natural killer cell level and activity, faster decline in CD4 count and increased risky sexual behavior that results in contracting additional disease that compromise the immune system [14–16].

The federal MoH of Ethiopia prioritizes health care delivery at the community level. However; little attention is given for those who are suffering from mental health problem especially in prisons. There is no enough data on the prevalence of depression and associated factors among HIV infected prisoners in Ethiopia. Lack of information about depression in HIV infected prisoners may be a factor that contributes to poor or inexistent mental health care at the prison. Therefore; this study was aimed to investigate the prevalence of depression and associated factors among HIV positive prisoners at Addis Ababa central prison.

## Main text

### Methods

#### Study design and participants

Institution based cross-sectional study was conducted at central prison in Addis Ababa. Addis Ababa central prison is one of the Federal prisons located in Akaki Kaliti sub city. A total of 490 HIV positive prisoners had HIV care follow up at ART clinic of the prison during data collection. Of which, 312 of them were on ART. There was no mental health service in the prison. Those HIV infected prisoners incarcerated for more than 3 months were participated in the study. Those seriously ill and not capable to communicate were excluded.

#### Sampling and sample size calculation

Sample size was calculated using single population proportion formula by assuming the prevalence to be 50% due to the absence of similar study in the area. The 95% confidence interval and 5% margin of error were considered. Finally 10% non-response rate was added to get total of 422 samples. Simple random sampling technique was used to select the study participants from May to July 2016.

#### Data collection technique and procedures

Face interviewer administered questionnaire was used to collect data. Trained nurses working in other health institutions were involved in data collection. Participants chart was also reviewed to document their WHO clinical stage, recent CD4 count and ART regimen. In order to avoid double count, charts were labeled with marker once the patient was interviewed. The Amharic version of internationally validated Patient Health Questionnaire (PHQ-9) was used to assess depression. The (PHQ-9) consists of 9 items with ‘0’ = not at all, ‘1’ = several days, ‘2’ = more than half a day and ‘3’ = nearly every day. It has a score of 0 to 29. Depression was considered using a cut point greater than or equal to five [17]. Social support was measured using Oslo‐3 Social Support Scale (OSS‐3). By adding the three items poor social support was considered when they scored 3–8 while a score of 9–11 and 12–14 were considered to have intermediate and strong social support respectively. Stigma was assessed using a 12 item HIV stigma scale that is feasible, reliable, and valid instrument to assess HIV-related stigma [18].

#### Data analysis

The investigators checked the filled questionnaires for completeness, cleaned and entered to EPI Info version 3.5.3 statistical software and exported to SPSS version 20 for analysis. Descriptive and summary statistics were used to explain the study population with respect to significant variables. Variables with *P* value less than 0.2 in the bivariate analysis were fitted to multivariable logistic regression model. Odds ratio with their 95% CI was calculated. Statistical significance was considered at P-value < 0.05 in multivariable logistic regression model.

### Result

In this study, a total of 400 study participants were involved with a response rate of 94.8%. The prevalence of depression among HIV infected prisoners was found to be 66.5% [95% CI 62, 71)]. The mean age of the respondents was 38.9 (SD ± 10.7) years. Majority 378 (94.5%) of the study participants were males. About one-third of the prisoners had primary in their educational status. Large proportion (67.3%) of prisoners disclose their HIV status to their close friends while only 4.5% of disclose to the media. More than half (56.0%) of the HIV positive prisoners had poor social support and (Table 1).

**Table 1:** Socio-demographic and Psychosocial characteristics of prisoners at central prison (Kaliti), Addis Ababa Ethiopia, 2016, (n = 400)

Variables	Category	Frequency	Percentage
Age (years)	18–27	64	16.0
	28–37	113	28.2
	38–47	145	36.3
	48–57	55	13.7
	58+	23	5.8
Sex	Male	378	94.5
	Female	22	5.5
Ethnicity	Amhara	180	45.0
	Oromo	98	24.5
	Tigray	64	16.0
	Gurage	18	4.5
	Welayta	22	5.5
	Others*	18	4.5
Marital status	Single	119	29.7
	Married	175	43.7
	Separated	25	6.3
	Divorced	42	10.5
	Widowed	39	9.8
Religion	Orthodox Christian	299	74.7
	Catholic	8	2.0
	Protestant	36	9.0
	Muslim	51	12.8
	Others**	6	1.5
Educational status	No formal education	61	15.3
	Primary	153	38.2
	Secondary	138	34.5
	Tertiary and above	48	12.0
Income	No income	117	29.3
	≤ 758 birr	73	18.2
	758–2150 birr	126	31.5
	≥ 2151 birr	84	21.0
Disclosure of HIV status	Yes	333	83.3
	No	67	16.8
Disclosure to partners	Yes	146	43.8
	No	187	56.2
Disclosure to parents	Yes	148	44.4
	No	185	55.6
Disclosure to friends	Yes	224	67.3
	No	109	32.7
Disclosure to medias	Yes	15	4.5
	No	318	95.5
Disclosure to others***	Yes	73	21.9
	No	260	78.1
Social support	Poor	224	56
	Moderate	141	35.3
	Strong	35	8.8
Perceived Stigma	Yes	176	44
	No	224	56

* Sidama, Somali **Wakefata, Jihovawitnes ***Relatives

More than one-third of prisoners were stayed in prison for 1–3 years. Twenty-eight (7.0%) of the prisoners had history of any psychiatric illness. Around 60% of study subjects were on WHO clinical stage I (Table 2).

**Table 2:** Prison related and clinical characteristics of prisoners at central prison (Kaliti), Addis Ababa Ethiopia, 2016, (n = 400)

Variables	Category	Frequency	Percentage
Length of stay in prison (years)	< 1	107	26.8
	1–3	149	37.3
	4–6	57	14.3
	7–9	52	13.0
	10+	35	8.8
Ever convicted	Yes	36	9.0
	No	364	91.0
How many times ever convicted	Once	19	52.8
	Twice	17	47.2
History of chronic illness	Yes	91	22.8
	No	309	77.2
History of psychiatric illness	Yes	28	7.0
	No	372	93.0
Family history of psychiatric illness	Yes	24	6.0
	No	376	94.0
Time since patient known his sero-status (years)	< 1	29	7.3
	1–3	69	17.3
	3–5	82	20.5
	> 5	220	55.0
CD4 count	≤ 500	62	15.5
	> 500	338	84.5
WHO clinical stage	I	241	60.2
	II	95	23.8
	III	56	14.0
	IV	8	2.0
On ART	Yes	254	63.5
	No	146	36.5
ART regimen	1c	38	15
	1d	25	9.8
	1e	136	69.3
	1f	15	5.9
Lost partner related to HIV	Yes	25	8.9
	No	255	91.1
Do you have children	Yes	275	68.8
	No	125	31.3
Children’s HIV status	Positive	23	8.4
	Negative	206	74.9
Ever hospitalized	Yes	78	19.5
	No	322	80.5

This study indicated that educational status, perceived HIV-stigma, social support, history of known chronic illness, WHO clinical stage and length of stay in prison were significant factors associated with depression. Prisoners with primary educational level were 4.17 times more odds of depression [AOR = 4.17, 95% CI (1.66, 10.16)]. Prisoners with perceived stigma due to HIV status were 3.9 times more likely to be depressed [AOR = 3.88, 95% CI (2.08, 7.25). Those having history of chronic illness (such Heart failure, Hypertension, Diabetes mellitus) were 2.9 times more odds of depression than those with no history of chronic illness [AOR = 2.88, 95% CI (1.34, 6.17)]. Those on WHO clinical stage II were 2.5 times more likely to be depressed compared to WHO clinical stage I [AOR = 2.47, 95% CI (1.19, 5.12)]. Prisoners who stayed for 4 up to 6 years in prison were 2.3 times more likely to be depressed [AOR = 2.27, 95% CI (1.22, 4.23)] and those who stayed for more than 10 years were 3.5 times more likely to develop depression than those who stayed less than 1 year [AOR = 3.53, 95% CI (1.15, 10.85)] (Table 3).

**Table 3:** Multiple Logistic regression analysis of depression among HIV positive prisoners at central prison (Kaliti) Addis Ababa Ethiopia, 2016, (n = 400)

Variables	Depression					
	Yes	No	COR	95% CI	AOR	95% CI
Educational status						
No formal education	43	18	2.39	1.085–5.260	3.03	0.902–10.205
Primary	107	46	2.33	1.199–4.514	*4.11*	1.66–10.19*
Secondary	92	46	2.00	1.026–3.898	2.28	0.923–5.624
Tertiary and above	24	24	1		1	
Social support						
Poor	162	62	0.55	0.351–0.855	1.14	0.419–3.098
Moderate	83	58	0.57	0.275–1.199	0.90	0.333–2.447
Strong	21	14	1		1	
Stigma						
Yes	144	32	3.76	2.364–5.987	*3.88*	2.08–7.25**
No	122	102	1		1	
History of chronic illness						
Yes	75	16	2.89	1.611–5.206	*2.88*	1.34–6.17**
No	191	118	1		1	
WHO clinical stage						
Stage I	148	93	1		1	
Stage II	69	26	1.67	0.991–2.806	*2.47*	1.19–5.12**
Stage III	44	12	2.30	1.157–4.589	2.10	0.90–4.88
Stage IV	5	3	1.05	0.245–4.486	1.23	0.19–7.67
ART						
Yes	158	38	1		1	
No	108	96	1.727	1.103–2.704	1.30	0.69–2.45
Length of stay in prison						
< 1 year	61	46	1		1	
1–3 years	100	49	1.54	0.921–2.571	1.949	0.91–4.20
4–6 years	46	11	3.15	1.473–6.750	*2.27*	1.22–4.23**
7–9 years	30	22	1.03	0.526–2.010	1.89	0.49–7.24
≥ 10 years	29	6	3.65	1.397–9.507	*3.53*	1.15–10.85*
Disclosure of HIV status to partners						
Yes	89	57	1		1	
No	137	50	1.76	1.103–2.791	1.64	0.93–2.88
Disclosure of HIV status to friends						
Yes	144	80	1		1	
No	82	27	1.69	1.010–2.820	1.54	0.84–2.84

NB: *P value < 0.05, * P value < 0.01

### Discussion

In our study the prevalence of depression among HIV positive inmates at central prison was found to be 66.5%, 95% CI (62%, 71%). Compared to studies in health care settings, this finding is higher. This may be due to the fact that majority of prisoners at central prison (Kaliti) were political prisoners and may not handled properly and safely. The finding is also higher than the finding of a study in prisons found in Northwest Ethiopia (43.8%) [19], southern Ethiopia (56.4%) [20] and southwest Ethiopia (41.9%) [21]. This may be due to the fact that the variation in the cut of point used to define depression in public health questionnaire (PHQ 9), variation in the prison setting i.e. central prison is a setting where political prisoners are incarcerated from every direction of the country. This finding was in-line with the study done in Uttar Pradesh (India) (67.3%) [22]. However, this finding is much higher than the lifetime prevalence of depression at Westville Correctional Center (24. 9%) [11]. The observed variation may be due to difference in study subjects. A study in Westville Correctional Center included all the prisoners irrespective of their HIV status and Mini International Nero-psychiatric Interview (MINI)] was a tool used to screen depression. Even the way handling inmates may have significant variation. Similarly, our finding is higher than another study conducted in South Africa [13] using Hospital Anxiety and Depression Scale (25.4%) and in Debre Markos referral hospital [23] using SRQ 20 to find (24.3%) of individuals living with HIV were screened positive for common mental disorder. This difference might be due to the fact that the socioeconomic variation in study subjects, difference in the way prisoners are handled at correctional institutions and the variation of tool used to screen depression. The finding of this study was higher compared to a study finding in USA (55.5%) [9] Taiwan (31.2%) [10], Westville Correctional Center (10.4%) [11], and North Carolina prison (44.3%) [24]. The observed discrepancy might be due to difference in socio-economic status between the study participants, clinical variation and dissimilarity of tools used to assess depression. The study in USA, Taiwan and North Carolina used Brief Symptom Inventory (BSI), Brief Symptom Rating Scale (BSRS-5) and Center for Epidemiological Studies-Depression Scale (CES-D) respectively.

This study revealed that, those HIV positive prisoners with primary in education were more likely to be depressed. This finding is supported with the finding in Guru Teg Bahadur Hospital Delhi, India [25] where uneducated HIV positive people were highly affected by depression than educated ones. However; another study also showed that education had no association with depression in HIV positives [22]. This discrepancy may be justified by the variation in tools used by these different studies. Center for Epidemiologic Studies-Depression (CES-D) and Montgomery-Asberg Depression Rating Scale (MADRS) were used in Guru Teg Bahadur Hospital and South-West part of Uttar Pradesh India respectively. Perceived stigma was positively associated with depression in HIV positive prisoners. This is agreed with a study in Jamaica [26], Debre Markos referral hospital [23] and South Africa [13]. This correlation may be due to the fact that people who perceive stigma may worry about themselves and become depressed. In this study previous history of chronic illness was associated with depression. The finding is consistent with the studies in USA and South Africa [9, 11]. This study also found that those on WHO clinical stage II were more likely to be depressed than WHO clinical stage I. It is supported by studies conducted in Debrebrihan Referral Hospital [27] and Califorinia [28]. This may be due to the fact that HIV positive prisoners with more advanced HIV disease are more likely to be diseased with opportunistic infections that cause depression.

This study indicated that age, sex, marital status, social support, history of diagnosis with psychiatric illness, CD4 count and term of sentence were not found to be statistically significant factors to depressive symptoms in HIV positive prisoners at central prison. It was inconsistent with a study conducted in Cameron [29], Harar [30] and India [25], which revealed depression were seen among participant with low CD4 count, unemployed, having poor social support, no or low family income and unmarried. This inconsistence may be due to the fact thatour study subjects where HIV positive prisoners. Whereas; the study subjects in Cameron, Harar and India were HIV positive non incarcerated people.. This study also showed that length of stay in prison is associated with depression. The odds of depression increased as the length of prison stay increased. This was supported by the study done in United States [31]. This may be due to feelings and disquieting when they stare what their life looks like after long time imprisonment.

### Conclusion

The finding of this study showed that prevalence of depression among HIV positive prisoners at central prison (Kaliti) was found to be high. Educational status, perceived HIV-stigma, history of chronic illness, WHO clinical stage and length of stay in prison were significant factors associated with depression. It suggests that there is a need to establish psychiatry care unit in the prison to screen and manage inmates. Special attention need to be given for those with primary educational level, advanced HIV status, who perceived HIV-stigma, with history of chronic illness and who stayed many years in prison. Further study with large sample size by including many prisons may be needed.

## Limitations

Some variables that may have associations with depression were not included (such as: frequency of family visit). Since this is cross-sectional study it is difficult to conclude the cause and effect relationship.

## Abbreviations

AIDS: Acquired Immune deficiency Syndrome
ART: antiretroviral therapy
BSI: Brief Symptom Inventory
BSRS: Brief Symptom Rating Scale
CES-D: Center for Epidemiologic Studies-Depression
EDHS: Ethiopian Demographic Health Survey
MADRS: Montgomery-Asberg Depression Rating Scale
MoH: Ministry of Health
SRQ: Self Reporting Questionnaire
WHO: World Health Organization
